# Emergency Physicians Ability to Recognize and Diagnose Opiate Use Disorder: A Qualitative Study

**DOI:** 10.7759/cureus.18216

**Published:** 2021-09-23

**Authors:** Christine Crain, Tracy Meyer, Devon Webster, Jacqueline Fraser, Paul Atkinson

**Affiliations:** 1 Dalhousie Medicine New Brunswick, Dalhousie University, Saint John, CAN; 2 Department of Emergency Medicine, Horizon Health Network, Dalhousie University, Saint John, CAN; 3 Emergency Medicine, Saint John Regional Hospital, Saint John, CAN; 4 Department of Emergency Medicine, Dalhousie University, Halifax, CAN; 5 Emergency Medicine, Horizon Health Network, Saint John, CAN; 6 Emergency Medicine, Dalhousie Medicine New Brunswick, Halifax, CAN

**Keywords:** substance use disorder, opioid agonist therapy, emergency department, harm reduction, opioid use disorder, opioid epidemic

## Abstract

Introduction

The opioid crisis is a significant public health problem for this generation. Proper treatment of patients with opiate use disorder (OUD) during vulnerable times is vital to their engagement in opiate agonist therapy (OAT). There is limited information as to the efficacy of ED practitioners in recognition of opioid withdrawal or OUD; this research was designed to fill this gap to advance our care of vulnerable populations.

Methods

Interviews were conducted with seven convenience-sampled ED physicians and nurse practitioners from the Saint John Regional Hospital by providing a clinical vignette. These one-on-one, scripted interviews, conducted by the principal and co-investigator, tell us about the ED physician’s understanding of OUD and withdrawal by posing questions around the presentation within the clinical vignette, as well as around general knowledge of OUD and acute withdrawal.

Results

All seven participants identified the patient in the case as being in opioid withdrawal but did not identify all symptoms in the vignette. Two correctly diagnosed our patient as having OUD based on the scene provided. Five physicians identified criteria that pointed toward this diagnosis but did not vocalize the connection. Only one discussed prescription of OAT as a treatment, most opting for symptom management and information on sites of self-referral for treatment. Finally, six of our interviewees expressed interest in prescribing buprenorphine but cited a need for more education around its use and initiation before feeling comfortable prescribing it.

Conclusions

While ED practitioners correctly recognize opiate withdrawal, there is a knowledge gap in the related diagnosis and evidence-based management of OUD. The development of education around these gaps will allow for stronger advocacy and better outcomes for this patient population.

## Introduction

The ongoing opioid crisis in Canada is one of the most significant public health problems of this generation; according to recent clinical practice guidelines, the mean national rate of hospital admissions related to opioid poisonings increased from nine hospital admissions per day in 2007/08 to more than 13 admissions per day in 2014/15. There are many factors that contribute to this public health emergency, such as stigma and lack of political will from municipal to federal levels for mental health services. There are several steps that physicians working in emergency departments can take to assist patients with opioid use disorder (OUD). Any increase in access to opiate agonist therapy (OAT) in New Brunswick requires a diverse toolset and approach. More information about these requirements is needed before any changes can be successfully made.

We do not yet know how effective Saint John Area Emergency Department (ED) physicians are in the diagnosis of opioid withdrawal or OUD. With the use of in-person interviews, we hope to gain a better understanding of ED practitioners’ ability to recognize and diagnose patients with an acute presentation of OUD. In addition, we hope to gain insights into current barriers to the provision of OAT from the ED.

This project, therefore, will determine ED physicians’ perceived ability to use OAT in their departments, prescribing practices, barriers and facilitators, and access to resources by conducting in-person interviews. The results will inform the implementation of model OAT initiation for the Saint John Regional Hospital Emergency Department.

Background

Opioid Withdrawal

Discontinuation of long-term opioids can produce withdrawal symptoms within hours of last use (e.g., four to six hours after last heroin use); the presence of these symptoms contributes to the clinical diagnosis of spontaneous opioid withdrawal. They typically fall into one of four categories that occur in a relatively predictable time frame. First, anxiety type symptoms occur within the first 8-12 hours of last use, exemplified by sympathetic and central nervous system arousal (e.g. mydriasis, mild hypertension and tachycardia, anxiety and irritability, insomnia, agitation, restless leg syndrome, general restlessness, tremor, and, less frequently, low-grade temperature and tactile sensitivity). Second, gastrointestinal distress (e.g. abdominal cramps, diarrhea, nausea, and/or vomiting) and flu-like symptoms (e.g. lacrimation, rhinorrhea, diaphoresis, shivering, and piloerection) occur over the following 24-36 hours, with increased bowel motility especially prevalent toward the end of this time frame. Other symptoms, such as yawning, sneezing, anorexia, dizziness, myalgias or arthralgias, and leg cramps, presenting throughout this withdrawal period, with most peaking 36-72 hours later [1].

Opioid Use Disorder

According to the Diagnostic and Statistical Manual of Mental Disorders, fifth edition, OUD is characterized by the presence of specific behaviours around the use of opioid drugs. It presents as clinically significant impairment or distress in two of the following areas over the past twelve months: larger amounts used over a longer period than was intended; persistent desire or unsuccessful efforts to cut down use; time is spent in activities necessary to obtain, use, or recover from the effects of the opioid; a strong desire or urge to use; use resulting in a failure to fulfill major role obligations; continued use despite having persistent or recurrent social problems; important activities are given up or reduced because of use; recurrent use in physically hazardous situations; continued use despite knowledge of having a problem; tolerance; withdrawal [2].

OAT in the Emergency Department

Previous research has shown that ED physicians may have negative attitudes toward patients with substance use disorders [3]. Moreover, the proper treatment of patients during vulnerable times is very important to the engagement of these patients with OAT. This is an opportunity that is often missed in the ED. Immediately following an overdose event, physicians should begin a conversation with patients around OAT or other treatment options. Buprenorphine can be initiated in the emergency department and improves addiction follow-up care. It also decreases overdose and all-cause mortality [4].

A model for initiation of buprenorphine in the ED has had some success after implementation in other jurisdictions thereby increasing access to OAT and referrals to other specialist support programs [4-6]. Support from ED practitioners in the practice of prescribing buprenorphine is required for similar models to be tested in Canada.

Prescribing Buprenorphine

As buprenorphine can be administered by trained physicians without special licencing, ED practitioners are in a special position to help these patients at a most sensitive time. They are often a common first point of contact with those patients who would benefit most from initiating OAT; therefore, it is of utmost importance that these physicians be ready and willing to help as needed.

## Materials and methods

With the approval of the Saint John Regional Hospital’s Research Ethics Board, one-on-one interviews were conducted by study investigators of seven total ED physicians and nurse practitioners from the Saint John Regional Hospital (SJRH). Practitioners were given a clinical vignette of OUD and withdrawal (see Appendix A for clinical vignette). They were then asked, based on the case, to identify their top diagnoses, as well as to provide examples of how patients presenting with OUD and withdrawal should be provided care in the emergency department and how that care was different in the past.

These participants were recruited as a convenience sample of volunteers from staff meetings, staff email, and in-person interactions with ED physicians. The interviews continued until there were no more novel ideas emerging from the discussions. Participation was voluntary, signed consent was obtained, and physicians were offered a $50 gift certificate as a ‘thank you’ gift for their time. The duration of the interviews was from 15-30 minutes, with a mean length of around 20 minutes.

The principal investigator (Devon Webster [DW]) and one co-investigator (Christine Crain [CC]) conducted interviews, using a script and advanced pilot interviews to create uniformity in style, thereby limiting variability between interview styles when conducting interviews with ED physicians (see Appendix B for interview script).

Interviews were directly recorded via Zoom H2n Handy Recorder (Zoom Corporation, Tokyo, Japan) then transcribed by interviewers to Microsoft Word (Microsoft Corporation, Redmond, Washington) files so that they could be shared between investigators. The transcription was uploaded to a password-protected version of NVivo. The results of the interviews were processed via thematic analysis to allow for the highlighting of repeated ideas and concepts. They were then downloaded to a password-protected laptop.

Interview transcripts were encoded using NVivo 12 Plus by the principal and co-investigators, as well as by SJRH research services to compare for similarity and to limit bias. These codes were then placed into categories to better understand their relationships and what they tell us about the ED physician’s understanding of OUD and withdrawal.

## Results

Recognition of withdrawal

Of the practitioners that were interviewed, all seven identified the patient in the case as being in spontaneous opioid withdrawal. All participants recognized these symptoms as stemming from opioid withdrawal (see Table 1).

**Table 1:** Count of criteria identified by interviewed physicians with percentage of those interviewed in brackets GI - gastrointestinal

Opioid withdrawal	Included by physicians
Gastrointestinal distress	
GI upset (nausea or vomiting)	6 (86%)
Flu-like symptoms	
Arthralgias	3 (43%)
Diaphoresis	6 (86%)
Rhinorrhea	3 (43%)
Sympathetic/central nervous system arousal	
Anxiety	6 (86%)
Dilated pupils	2 (29%)
Piloerection	1 (14%)
Restlessness	0
Tachycardia	7 (100%)
Tremor	6 (86%)
Yawning	0
Opioid use disorder	
Larger amounts used over a longer period than was intended	5 (71%)
Persistent desire or unsuccessful efforts to cut down use	2 (29%)
Time is spent in activities necessary to obtain, use, or recover from the effects of the opioid	0
Strong desire or urge to use	0
Use resulting in a failure to fulfill major role obligations	2 (29%)
Continued use despite having persistent or recurrent social problems	0
Important activities are given up or reduced because of use	0
Recurrent use in physically hazardous situations	1 (14%)
Continued use despite knowledge of having a problem	0
Tolerance	7 (100%)
Withdrawal	7 (100%)
Incorrect inclusions	
Dishevelled appearance	1 (14%)
History of psychiatric illness	1 (14%)

Diagnosing OUD

Of the physicians that were interviewed, only two correctly diagnosed our patient as having OUD based on the clinical vignette provided. Five other physicians identified criteria that pointed toward the clinical diagnosis of OUD but did not vocalize this connection. Meanwhile, when questioning led to other symptoms of OUD, there were many that were not included by any interviewees as well (see Table 1). Specifically, continued use despite personal or social problems, a desire to use, and important activities being given up were not discussed by anyone. Additionally, there were some incorrect inclusions or assumptions made by interviewees with regard to what is involved in the diagnosis of OUD. One such physician included “he appears dishevelled, so he appears unkempt,” and another that a “history of psychiatric illness” would also be included in their clinical criteria.

Barriers

Finally, six of our interviewees expressed interest in prescribing buprenorphine from the ED but had not yet, for various reasons. The most commonly cited reasons, by five of our interviewees, for not prescribing buprenorphine from the ED were a need for more and repeated, education sessions around its proper use and initiation. As well, there were requests for a community bridge to make sure these patients are not lost to follow-up. Other suggestions to improve access for patients with OUD were having an order set or an algorithm available and easily accessible to ED clinicians to make starting buprenorphine simpler.

## Discussion

While all physicians recognized that the current presentation reflected a spontaneous opioid withdrawal syndrome, knowledge of some specific symptoms was lacking. Neglect of rhinorrhea and/or arthralgias as withdrawal symptoms may lead to missed opportunities for discussion of OAT. If a patient is presenting with these symptoms and the physician fails to identify the connection to opioid withdrawal, then the opportunity to engage in conversation with the patient around treatment options, such as OAT, may be missed. Along these lines, Wiercigroch et al. describe missing subtle presentations of withdrawal as a common barrier to the proper treatment of OUD [7].

The education component for increasing buprenorphine prescription from the emergency department was brought up by nearly all participants. This should help with the correct identification of OUD, as most did not vocalize the connection of spontaneous withdrawal to clinical OUD. In a setting where symptom management appears to be the preferred method of treatment, this represents a significant missed opportunity to discuss other treatment options, such as OAT. These missed criteria for OUD reflect the need to educate practitioners, especially those missing the OUD diagnosis, on the criteria required to screen patients. By increasing screening, we should be able to also increase patient enrollment in OAT and other harm reduction strategies.

Additionally, education should help reduce withdrawal/OUD diagnosis based on inappropriate criteria or assumptions as discussed with the patient when they are being seen as there are many reasons a patient may appear “dishevelled”. Using this as a part of clinical criteria for OUD reflects the bias some physicians may have towards these patients [3]. The education component should also help reduce the stigma surrounding co-occurring conditions; while psychiatric illnesses often co-occur with use disorders, it is important to recognize these are not a part of OUD but as psychiatric comorbidities that require individual treatment outside of OAT. The presence of these comorbid conditions may make treatment of withdrawal more difficult, but there are no studies to assess the effect of these comorbidities on the course or severity of opioid withdrawal [8].

Limitations

With the use of convenience sampling, these results may be limited by selection bias. Subsequently, ED physicians who are already interested in harm reduction and OUD may have provided opinions different from those of the rest of the department. Physicians also knew the topic was on opioids prior to interviews which may have helped identify opioid withdrawal as an initial diagnosis. The small sample size also reduces the ability to extrapolate the data to the rest of the emergency department or even to other clinicians. Additionally, the size of our site, with only about 60,000 patients visits per year, may make these results less generalizable than studies performed at larger or more sites.

## Conclusions

Investigation into the ability of these clinicians' ability to diagnose and manage OUD in the ED will provide guidance for the future development of protocols and education for the optimization of patient care when presenting with OUD in SJRH ED, both of which were requested by many of the physicians interviewed. As only two of the participants were able to correctly diagnose OUD in the vignette, this is an area where improvement will be relatively easy to obtain.

The need for further education around the initiation of buprenorphine in the ED is another gap that was identified by six of our seven participants. While this can be addressed alongside the above-mentioned education for OUD, implementation of order sets for clinicians to use will make the process more streamlined even among clinicians that feel in need of guidance in the presentation of OUD and withdrawal.

There will be the development of education around the opinions and knowledge gaps of diagnosis and management of OUD and spontaneous opioid withdrawal as presented above. This will allow for better advocacy for patients and for changes within the community itself for a better harm reduction pathway such as a strong community bridge and changes to existing or the creation of new algorithms and protocols for the induction of patients into OAT.

## Appendices

Appendix A: Clinical vignette

Joe is a 30-year-old man presenting to your emergency department complaining of nausea. He has been vomiting since yesterday morning and on exam, he appears anxious and tremulous. He appears dishevelled and admits to you that he often has difficulty making ends meet. He wipes his nose again as you check his vitals and find that he is tachycardic with a heart rate of 102.

Two and a half years ago he was involved in a motor vehicle collision and developed significant low back pain for which he was started on Percocet. Eventually, he found these were not effective at controlling the pain and a friend provided him with leftover hydromorphone from previous surgery. Joe’s family doctor was unwilling to provide him further prescriptions and since then, he has been getting hydromorphone from a friend of a friend, despite the high cost. He has been requiring gradually increasing doses to keep the pain at bay and whenever he tries to decrease the amount or runs out, he feels nauseous and finds the pain unbearable. He recently ran out of his hydromorphone and had to miss work again. He continues to feel unwell today and is asking you for hydromorphone.

Appendix B: Interview script

Interviewee demographics:

Type of practitioner: EM+1/5 yr EM/NP/GP

Years practicing and locations:

Can you tell me a bit about what your specific interests in emergency medicine are?

Case: (To be handed to interviewees on a separate form)

Part 1: Ability to recognize opioid use disorder and opioid withdrawal

1. “Name your primary and secondary diagnoses from this case.”

Any other diagnoses on your differential? (Particularly if they have not specifically mentioned OUD/withdrawal)

2. “What features in the case are suggestive of opioid use disorder? What other diagnostic features are you aware of?”

If they mention ++symptoms of withdrawal, mention that together they form the constellation of withdrawal and thus a single part of the OUD diagnosis. Have them expand on the OUD symptoms ASIDE from withdrawal.

3. “What features, in this case, are suggestive of opioid withdrawal? What other signs or symptoms not described are suggestive of opioid withdrawal?”

4. Do you know of any resources that may help you stratify a patient’s degree of withdrawal (e.g. similar to CIWA)?

Part 2: Understanding perceptions of opioid use locally

5. “Do you feel that opioid misuse is a problem in Saint John? Why or why not?”

Can you expand on the ways that OUD has presented to the ED in your experience? (E.g. overdose, injection-related infection (IE, abscess), mismanaged chronic pain, other)

Do you often see affected people in the community or outside of work?

Is it in the news or other media outlets here?

6. “How often do you encounter patients who misuse opioids in your practice? Every shift, couple of shifts or less?”

Part 3: Treatment of opioid use disorder

7. “What type of treatments have you provided to patients similar to Joe in the past?”

With respect to:

Overdose?

Withdrawal?

Primary and secondary prevention: take-home naloxone, bloodborne pathogens, information on needle exchange facilities, shelters, education on safe injection or other harm reduction measures (e.g. using PO vs IN or injection)

8. “What type of referrals have you provided to patients similar to Joe in the past?”

9. “Tell me about your understanding of buprenorphine.”

Are you familiar with the indications for buprenorphine?

What is your understanding of the ‘naloxone’ component?

Part 4: Barriers to starting buprenorphine in the emergency department

10. “In terms of harm reduction care, what do we do well for patients with opioid use disorder from our emergency department? (With respect to OUD or addictions in general)”

Cultural and work attitudes?

Management?

Continuing medical education and rounds coverage?

Connecting with resources? Like what?

Providing helpful harm reduction education for pt’s with OUD?

Screening for comorbidity?

Education on safe use?

Protocols?

11. “What ways could we improve our delivery of harm reduction care at our center? (With respect to OUD or addictions in general)”

Cultural and work attitudes?

Management?

Continuing medical education and rounds coverage?

Connecting with resources? Like what?

Providing helpful harm reduction education for pt’s with OUD?

Screening for comorbidity?

Education on safe use?

Protocols?

9. “Would you be willing to start a patient presenting to our emergency department on buprenorphine? Why or why not?”

If we had a protocol in place would this make you more likely to initiate it?

If we had established community follow up would you?

12. “What barriers, if any, make it difficult to start buprenorphine from our emergency room?”

13. “Is there anything that could be done to help remove these barriers?”

End of the interview:

Provide relevant resources if physicians are interested in getting further information (e.g. COWS, start protocols)

If you feel its appropriate, consider challenging ideas that aren’t compatible with current research (e.g. EDs that provide buprenorphine are likely to be bogged down with patients seeking ‘detox’)
